# Hair follicle organoids: advances in construction, applications, and translational challenges

**DOI:** 10.3389/fcell.2026.1836905

**Published:** 2026-05-28

**Authors:** Xinyi Yao, Rui Ding, Yuan Chuanjian, Duorong Zeng, Yang Liu, Jun Zhang, Junling Zhang

**Affiliations:** 1 Department of Dermatology, Tianjin Academy of Traditional Chinese Medicine Affiliated Hospital, Tianjin, China; 2 Graduate School, Tianjin University of Traditional Chinese Medicine, Tianjin, China

**Keywords:** hair follicle organoids, hair follicle stem cells, organoids, regenerative medicine, stem cells, tissue engineering

## Abstract

Hair follicles (HFs) are complex mini-organs characterized by a highly organized structure and cyclic regeneration. In recent years, hair follicle organoids (HFOs) have emerged as promising three-dimensional *in vitro* models that partially recapitulate the architecture and function of native hair follicles, providing new opportunities for studying hair biology and related disorders. This review first outlines the key biological features of HFs and highlights the essential role of epithelial–mesenchymal interactions in folliculogenesis, which serve as the foundation for organoid construction. We then summarize the fundamental principles of organoid technology and systematically compare current strategies for HFO generation, including primary cell-based co-culture systems and induced pluripotent stem cell (iPSC)-derived approaches, with an emphasis on their advantages and limitations. Furthermore, we discuss the applications of HFOs in disease modeling, drug screening, and regenerative medicine, while critically addressing current challenges such as difficulties in large-scale cultivation, limited maturity, and lack of standardization. Overall, HFOs represent a promising platform bridging basic research and clinical translation. Future efforts integrating biomaterials, microfluidic systems, and bioengineering technologies are expected to enhance their physiological relevance and accelerate their translational potential.

## 1 Introduction

The hair follicle (HF) not only is responsible for the cyclic growth and regeneration of hair but also participates in complex physiological processes such as skin barrier function, sensory function, pigmentation, and local immune regulation. Hair follicle organoids (HFOs) not only have the potential to more accurately simulate the processes of their development, cycle regulation, and pigment generation, but also provide a potential platform for the analysis of disease mechanisms, high-throughput drug screening, and hair follicle regeneration. However, to date, HFOs are generally characterized by limited model maturity, difficulties in long-term function, and the absence of standardized regulations. There is a significant gap between the *in vitro* models and clinical translation, and these issues all require systematic sorting and evaluation.

## 2 Biological characteristics of hair follicles

### 2.1 Mechanism of hair follicle growth and development

The hair follicle has a complex physiological structure. It is formed by epithelial cells derived from the epidermis and mesenchymal cells derived from the dermis through epithelial–mesenchymal interactions (EMI), and it achieves periodic growth and regeneration through this interaction.

Hair follicles are formed in the embryonic development stage, and their development begins in the third month of pregnancy. The development of hair follicles is roughly divided into three stages: formation of the follicular placode, follicular morphogenesis, and cell differentiation with function (Cao et al., 2025). First, mesenchymal cells in the dermis are activated to secrete Wnt signaling molecules; upon sensing these signals, epidermal cells proliferate locally and densely, thereby determining the location of hair follicles and forming a hair placode (Zhao et al., 2021). The Wnt/β-catenin and EDA/EDAR/NF-κB signaling pathways play a key role in this process, not only initiating hair follicle generation but also participating in maintaining the stability of the primary hair follicle placode. The hair placode expresses signaling molecules such as FGF20, which act on the underlying mesenchymal cells, promoting their proliferation and survival (Vázquez-Ulloa et al., 2022). The Notch signaling pathway maintains the balance between self-renewal and differentiation, induces stem cell pluripotency to maintain gene expression, and inhibits premature differentiation, thereby ensuring stem cell pool regeneration and normal circulation.

Furthermore, under the induction of Sonic hedgehog (Shh) signaling pathway, keratinocyte proliferation indirectly drives the hair placode to extend downward and agglutinate, and the BMP signal around the condensate is increased, which is beneficial to the development of keratinocytes, it participates in the formation of hair follicles and inhibits the induction of hair follicles by adjacent epidermal cells to prevent further enlargement of hair follicles. Dickkopf-related protein 4 (DKK4), as an inhibitory signaling molecule of Wnt signaling pathway, regulates the proliferation and differentiation balance of hair follicle epithelial cells. The dermal Noggin signaling protein plays a critical regulatory role as a BMP antagonist by inhibiting BMP signaling and regulating transcription factors such as Lef1 to control hair follicle epithelial induction. Dermal papilla cells (DPCs) are formed by wrapping dermal condensates with epithelial cells, establishing the initial structure of hair follicles (Ge et al., 2020). FGF7 and FGF10 signals from dermal papillae further stimulate epithelial cell proliferation, promoting hair follicle growth and extension in a “concentric circle” arrangement. During this process, the hair follicle placode cells can generate various types of cells and provide stem cell sources such as the bulge region’s multipotent stem cells for the periodic regeneration of the hair follicle (Sulic et al., 2023).

Finally, the hair follicle epithelium differentiates into various cellular substructures of the hair follicle. The hair follicle epithelial cells wrap around the dermal papilla to form a hair bulb, and the surrounding epithelial cells differentiate into an outer root sheath. The hair matrix cells near the dermal papilla proliferate and differentiate to form a hair shaft and an inner root sheath, sebaceous glands and arrector pili muscle and other ancillary structures also gradually developed, forming a complete function of the mature hair follicles. The mature hair follicle epithelium is composed of stromal keratinocyte, inner root sheath and outer root sheath. The stromal keratinocyte is an actively proliferating cell within the hair follicle that contributes to the formation of the hair follicle axis. In addition, neural crest cells originating from the ectoderm undergo directed differentiation to form melanocyte stem cells, which are localized in the bulge region of the hair follicle, continuously providing cellular sources for the hair follicle pigment system. The pigmentation of the hair shaft is regulated by mature melanocytes located near the matrix cells, resulting in a specific pigment phenotype for the new hair shaft (Vatanashevanopakorn and Sartyoungkul, 2023).

### 2.2 Cyclical regulation of hair follicles

The cyclical process of hair follicle regeneration, including anagen, catagen, and telogen. In anagen phase, the cells proliferated. In catagen phase, the hair follicles retracted and the cells were mainly apoptotic. In telogen phase, the cells remained relatively quiescent. After telogen, the cells were activated, enter anagen again. The mechanism of human hair follicle cycle is not as clear as that of mice. This periodic change is a distinctive feature of hair follicles that distinguishes them from other organs. The hair follicle regulation mechanism involves complex interactions among multiple signaling pathways and cytokines, including the Wnt/β-catenin, BMP, and Shh pathways.

Wnt/β-catenin signaling is the main initial signal for the hair follicle cycle to enter Anagen, and its activation can promote the activation of hair follicle stem cells (HFSCs), the proliferation of hair stromal cells, and hair follicle growth. At the same time, Shh signaling pathway promotes the proliferation and survival of hair stromal cells, and further promotes the proliferation and morphological construction of hair follicle epithelial cells. As the anagen phase approaches its end, apoptosis-related signaling pathways such as TGF-β/BMP are activated, causing the hair follicle to transition into the catagen phase. Hair matrix cells stopped proliferation and gradually apoptosis, hair follicle structure retracted upward, hair follicle length shortened. The initiation of catagen involves a shift in multiple signaling pathways, including the upregulation of BMP and FGF5 signaling and the decline in Wnt and Shh activity, which collectively trigger the apoptotic program, contributing to the structural degeneration of hair follicles (Ceruti et al., 2021). After the end of catagen, the hair follicle enters telogen. At this time, the stem cells in the bulge area of the hair follicle are in a relatively quiescent state, the volume of dermal papilla cells is reduced, and the secretion of signaling molecules is reduced. Telogen maintenance is associated with sustained activation of the BMP signaling pathway and inhibition of Wnt signaling (Liu et al., 2026). When the transition occurs from telogen to anagen, DPCs are reactivated and secrete signaling molecules such as Wnt and FGF, stimulating the proliferation and differentiation of bulge stem cells to initiate a new hair follicle growth cycle (Mehta et al., 2025). These pathways cross-affect cell populations in different regions of the hair follicle and mediate the hair follicle cycle. In addition to direct molecular signaling pathways, hair follicle growth is also affected by a variety of endogenous and exogenous factors. Endogenous factors include genetic background, hormone levels, and metabolic status, among which the role of androgens is particularly crucial (Burshtein et al., 2026); while exogenous factors include nutritional intake, hormone levels, neural regulation, drug interventions, and environmental pollution (Wang et al., 2026).

## 3 Basic principles and application advantages of hair follicle organoid technology

With the progress in cell isolation techniques, Worst and colleagues demonstrated that neogenesis of HFs occurred only when epidermal cells were transplanted alongside dermal cells (Worst et al., 1982). Researchers have gradually realized that there are obvious limitations in traditional two-dimensional cell models in hair follicle research, for example, the single cell type, the rapid loss of the expression characteristics of hair follicle-induced genes, and the inability to truly simulate the complex epithelial-mesenchymal interaction of hair follicles. These issues have been bottlenecks in hair follicle regeneration research. At the same time, although animal experiments have certain *in vivo* relevance, they are limited in application due to high costs, species differences, and ethical issues. In this context, organoid technology has gradually become an important model in hair follicle biology research because of its ability to highly simulate the three-dimensional structure and function of human organs.

Most organs can be partially reconstructed by simulating embryonic conditions and utilizing related morphogens and extracellular matrix proteins (de Groot et al., 2021). In 2009, Sato et al. (2009) successfully cultured mouse intestinal organoids from single LGR5 adult stem cells *in vitro* for the first time and established an organoid culture system. Subsequently, more organoid models were developed, including liver organoid, pancreas organoid, kidney organoid and so on.

Hair follicle organoids are miniature tissues formed by the self-assembly of hair follicle-derived stem cells or precursor cells (such as DPCs, keratinocytes, HFSCs, etc.) in an *in vitro* three-dimensional co-culture system, through cell-cell and cell-extracellular matrix (ECM) interactions. The core principle is to use the ECM (such as Matrigel) to provide scaffold support, and add specific growth factors, signaling molecules and nutrients to simulate the microenvironment of embryonic hair follicle development, promoting cells to proliferate, differentiate, and spatially arrange in a manner similar to *in vivo* organogenesis, ultimately forming organoid structures with hair follicle bud-like structures and even complete hair shafts.

Various types of hair follicle-related cells are included in the hair follicle organoid models, which better retain the functional characteristics of the original tissues and form a more complex intercellular biological communication and signal regulation network (Kageyama et al., 2026). In this network, cells achieve mutual influence, feedback regulation, and coordinated development through paracrine, autocrine, and other mechanisms. Cell-cell interactions and cell-extracellular matrix interactions support the establishment of cellular microenvironmental homeostasis, contribute to the formation and maintenance of micro-tissue organs with specific spatial structures and functions (Liang et al., 2026a). The organoid model has a more stable genome, enables long-term stable culture *in vitro* and maintains the stability of the genetic background, and is suitable for high-throughput, large-scale screening, and can be used as a tool for future research, and can accurately simulate the cellular heterogeneity, structure, and physiological functions of primary tissues (Bajouri et al., 2025). At the same time, the operation of organoid culture systems is relatively simple, which can effectively reduce the use of animal experiments, lower research costs, and shorten the development cycle (Mostina et al., 2025). These advantages make organoid technology play an important role in many fields, such as disease model construction, drug efficacy screening and safety assessment, organ transplantation, and regenerative medicine (Corrò et al., 2020). It provides an unprecedented *in vitro* experimental platform for the study of hair follicle development mechanisms and the treatment of hair loss regeneration.

## 4 Methods for construction of hair follicle organoids

**FIGURE 1:**
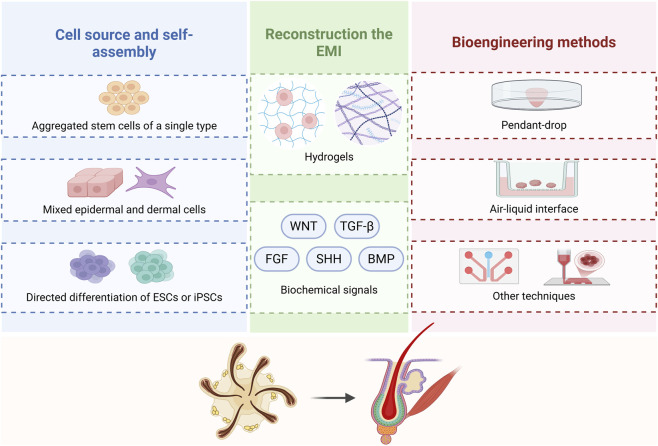
The main construction strategy of hair follicle organoids. This schematic diagram systematically illustrates the primary workflow for generating hair follicle organoids, divided into three interconnected modules. The left panel (blue) summarizes cell sources and self-assembly approaches, including aggregation of single-type stem cells, co-culture of mixed epidermal and dermal cells, and directed differentiation of embryonic stem cells (ESCs) or induced pluripotent stem cells (iPSCs). The central panel (green) emphasizes the reconstruction of epithelial–mesenchymal interactions (EMI) through hydrogel scaffolds and key biochemical signaling pathways. The right panel (pink) presents bioengineering methods employed for HFO assembly, including pendant-drop culture, air-liquid interface (ALI) culture, and other advanced techniques. The bottom section depicts the transition from a simplified hair follicle germ-like structure to a mature hair follicle with associated appendages (such as sebaceous glands and arrector pili muscle), highlighting the ultimate goal of achieving functional hair follicle regeneration.

For the hair follicle, a small organ which highly dependent on epithelial–mesenchymal interactions and cyclic regulation, the organoid strategy also shows potential, but also faces unique challenges - how to reproduce the induction ability of DPCs, the cyclic activation of bulge stem cells, and the complete process of hair shaft-pigment formation *in vitro* (Zhou et al., 2025). Based on the above issues, in recent years, researchers have developed various construction strategies for hair follicle organoids (HFOs). It can be clearly seen in Figure 1.

HFOs are usually composed of epithelial cells, mesenchymal cells, or stem cells as seed cells, combined with the extracellular matrix to simulate the microenvironment of hair follicle development *in vivo*, to reconstruct EMI, and to provide a theoretical basis for the development of hair follicle organoids, to achieve cell self-assembly and functional differentiation (McElwee and Sundberg, 2025). Hair follicle keratinocyte (hair follicle epithelial stem cell) is the main source of hair follicle epithelial cells. Co-culturing them with DPCs to induce EMI is an important prerequisite for the formation of hair follicle organoids. DPCs are the signal center of the hair follicle, which, through the Wnt, Shh, TGF-β, and other signaling pathways, cascade to affect the adjacent HFSCs and are the key components mediating hair follicle growth and cycle regulation (Ji et al., 2021).

The initial *in vitro* skin models consisted of a single layer of differentiated epidermis. Under conventional 2D culture conditions, follicle lineage-related cells adhered to the surface of the monolayer cell culture. At this stage, the cells experienced non-physiological mechanical constraints, leading to altered intercellular interactions and disrupted microenvironmental signaling. After passaging, these cells often lost their ability to induce and regulate hair follicle differentiation, presenting significant limitations (Topouzi et al., 2017). Subsequently developed full-thickness skin models, constructed by keratinocyte seeding on extracellular matrix scaffolds, or using fibroblasts to secrete extracellular matrix themselves, have been used to investigate the role of the extracellular matrix in skin regeneration, with the three-dimensional support of extracellular matrix and dynamic mechanical signal, the primary simulation of epidermis and dermis can be realized.

### 4.1 Cell source and acquisition

The cell source of HFOS was mainly generated by primary cell isolation culture (direct acquisition of hair follicle-associated cells from *in vivo*), pluripotent stem cell-directed induced differentiation, and cell co-culture (epithelial-mesenchymal cell co-culture producing cells). Although primary cell isolation and culture can directly obtain the target cell type, its source is often limited by ethical regulations, the difficulty of sample acquisition, and individual donor differences. For example, the acquisition of human DPCs often depends on invasive procedures such as scalp biopsy, and it is common for cell viability to decrease, phenotypes to become unstable, and function to decline after passage, which limits its promotion in large-scale experiments and clinical applications. Cell reprogramming technology has greatly expanded the cell sources of HFOs. In 2006, the Yamanaka team successfully induced the first induced pluripotent stem cells (iPSCs), the cornerstone of modern stem cell research (Takahashi and Yamanaka, 2006). Reprogrammed iPSCs possess multi-lineage differentiation potential and can be staged through regulated differentiation conditions to control cell fate, thereby constructing complex organoids containing multiple cell types. The main strategy for inducing the construction of skin and hair follicle organoids involves first directing iPSCs to differentiate into keratinocytes and fibroblasts separately, followed by co-culturing these 2 cell types. Under the synergistic action of specific signaling molecules, a layered structure resembling skin is formed.

Different cell sources each have their advantages and limitations. The choice of which cell source to use for constructing hair follicle organoids must comprehensively consider various factors such as research objectives, experimental costs, ethical requirements, and subsequent application scenarios. Directed differentiation of iPSCs not only avoids ethical controversies but also enables unlimited cell expansion. Furthermore, since the cells are derived from the patients themselves, there is hope for reducing the risk of immune rejection in future regenerative medicine transplantation applications. However, this currently faces challenges such as low differentiation efficiency and difficulties in simulating signaling regulatory networks (Liang et al., 2026b).

### 4.2 Co-culture assembly of hair follicle germ

Co-cultured hair follicle germ formation is achieved by separating epithelial cells and DPCs and simulating the early assembly process of hair follicle germs through direct co-culture of epithelial and mesenchymal cells *in vitro*, mimicking the developmental stage of hair follicles during embryogenesis. Different cells were mixed and inoculated in a certain proportion, and under specific culture conditions, they were induced to recognize each other, adhere to each other and assemble into a polar hair follicle germ structure, which was then implanted into the body or a 3D culture system, HFOs including hair shaft and root sheath can be further developed. The core of the co-culture system lies in the dynamic interactions and signaling communication between epithelial cells and mesenchymal cells. This strategy implemented on platforms such as hanging drop or air-liquid interface culture can more accurately replicate the early stages of follicle development, simulate cell-cell interactions during the initial phase of follicle development, and enhance the structural regularity of organoids.

In addition to the types of cells derived from hair follicle-associated cells, the Fukuda team also studied the role of endothelium cells (ECs) and human adipose-derived stem cells (hASCs) in making hair follicle tissue grafts. ECs are localized in the dermal papilla region, and hair follicle germs (HFGs) containing endothelial cells exhibited higher expression of hair morphogenesis-related genes *in vitro* (Kageyama et al., 2021). When DPCs, mouse embryonic epithelial cells, and hASCs are mixed in suspension and form aggregates, they develop into dumbbell-shaped HFGs. hASCs are located on the side of the DPC aggregates, and the involvement of hASCs significantly enhances the expression of genes associated with hair morphogenesis (Nakajima et al., 2021). Although these studies lacked results on long-term survival as well as hair quality, and the studies were mixed with mouse embryonic epithelial cells and hASCs or hDPCs, there were heterogeneous cell combinations across species. However, these studies still reveal that the combination of growth factors and microenvironmental regulation significantly affect the maturity of organoids. The HFOs construction system established by controlling these interactions lays a key technical foundation for the subsequent application of hair follicle tissue engineering.

### 4.3 3D aggregation and microenvironment optimization

Based on the above co-culture framework, 3D aggregation technology initiates cell self-assembly by physical/chemical means. Early HFOs studies mainly used co-culture of embryonic or neonatal mouse dermal cells and epithelial cells, and used the hanging drop method to establish a microenvironment model (Bauer et al., 2022).

The follicle reconstruction system was first established by Rogers et al. (1987). After isolating and purifying follicle-associated cells from mouse dermis, these cells were cultured in type I collagen and recombined with dermal fibroblasts, then transplanted onto the backs of mice to successfully induce hair regeneration. Kang et al. (2012) observed the initial formation of hair follicles after mixing 3D DPCs cultured by the hanging drop method with neonatal mouse epidermal cells, thereby first confirming the feasibility of the hanging drop method in HFOs construction. The hanging drop method can control the formation of uniform 3D cell spheroids through surface tension and the interaction between surface tension and gravitational field, but precise control of spheroid size and subsequent expansion remain challenging. Higgins et al. (2013) further achieved *in vitro* expansion of DPCs using the hanging drop, while constructing a local microenvironment capable of inducing hair follicle formation, partially restoring the dermal papilla gene expression profile (e.g., SOX2, BMP4) and correspondingly recovering the hair-inducing properties of DPCs. Although the hanging drop method facilitates initial aggregation, its lack of spatial control and scalability limits its translational potential compared with scaffold-based systems. Weber et al. (2019) utilized the hanging drop to combine neonatal foreskin keratinocytes with scalp dermal cells, observing the formation of hairpin-like structures at the epidermal cell protrusions where dermal cells aggregated, thus establishing the first example of *in vitro* differentiated HFOs; however, the dermal cells cultured using this method could not be successfully expanded. Air-liquid interface (ALI) stereoculture can simulate the natural environment, promote cell self-organization and directional differentiation based on lineage type, and promote the growth of the number and structure of hair follicles in organoids (Sun et al., 2025). This method is relatively straightforward, but compared with the hanging drop, it is inefficient and costly, and the constructed organoids still need to be optimized in terms of structural integrity and functional maturity.

Multiple teams are exploring novel cultivation systems to further optimize the microenvironment. Optimizing cell seeding density and culture time can reduce cell apoptosis and improve organoid formation efficiency. Adjusting the composition and stiffness of the extracellular matrix can provide an appropriate physical scaffold for spatial arrangement and interactions among cells, making it closer to the *in vivo* microenvironment during hair follicle development. Su et al. (2019) prepared a suspension by mixing fetal scalp dermal progenitor cells and adult foreskin epidermal stem cells (Epi-SCs) at a ratio of 2:1, and added recombinant WNT3a protein to the culture medium, resulting in the formation of numerous HFOs within 24 h, significantly improving the construction efficiency. Meanwhile, the study found that *in vitro* pre-aggregation-initiated interactions between epidermal and dermal progenitor cells, leading to activation of the Wnt pathway and the development of dumbbell-shaped structures termed “Type I aggregates.” Liu et al. (Liu et al., 2023) constructed a 3D co-culture system by encapsulating DPCs in Matrigel, followed by co-culture with epithelial cells, which further enhanced the expression of key genes associated with hair regeneration (such as SOX9, LEF1, and β-catenin).

Fukuda et al. (Kageyama et al., 2018) formed HFG spherical structures—where epithelial cells are inside and mesenchymal cells are outside—by resuspending mixed cultures in DMEM/F-12 medium supplemented with 2% low-concentration matrix gel, instead of forming dumbbell-shaped structures. HFGs secrete factors that promote the expression of β-catenin and CD133 in hair follicles, significantly enhancing hair shaft production, revealing the critical influence of matrix gel concentration on cellular spatial arrangement through microenvironment modulation (Hu et al., 2020). The team initiated self-assembly in low concentrations of Matrigel, forming a core-shell structure of mesenchymal cells surrounding epithelial cells. During subsequent cultivation, hair shafts grew straight within the basement membrane matrix (Matrigel) and developed pigmented regions, yielding mature hair shafts approximately 3 mm in length by day 23 (Kageyama et al., 2022). When transplanted onto the backs of nude mice, they demonstrated robust *in vivo* hair follicle regeneration activity and enabled monitoring of melanosome production and transport, providing an ideal model for studying hair follicle development mechanisms (Nakajima et al., 2021).

### 4.4 Complex structure construction

Based on the iPSCs technology, once the cells are successfully transformed into multipotential stem cells, they can be further induced to differentiate into specific types of cells. The Koehler team used TGF-β inhibitor (SB431542) and recombinant BMP4 to induce iPSCs to differentiate into both ectodermal and mesodermal cells, which then self-assembled into epidermal and dermal chimeric structures. Then treated with FGF2 and BMP inhibitor (LDN-193189), successful construction of iPSCs-derived mouse HFOS in 3D culture confirms the feasibility of this construction method (Lee et al., 2018; Klingenstein et al., 2025). In 2020, the team used iPSCs to generate 3D skin organoids containing hair follicles, nerves, and sebaceous glands. When transplanted into nude mice, these organoids successfully produced hair growing outward, directly validating the *in vivo* functionality of human iPSC-derived hair follicle organoids (Lee et al., 2020).

In 2022, Ramovs et al. (2022) optimized the culture system, and about day 60, hair germs were observed using iPSCs. By approximately day 130, stratified epidermis, hair follicles, and other skin appendages were differentiated, and the entire interfollicular epithelium was reconstituted, with structures and functions closer to human skin. In the same year, Lee et al. (2022) further confirmed that skin organoids induced from human iPSCs can differentiate into complex structures containing complete skin tissues (epidermis and dermis), as well as hair follicles, sebaceous glands, and sensory neurons around day 130, with a developmental stage similar to that of a 18-week-old fetus. In 2025, Mostina et al. (2025) further integrated iPSC-derived vascular organoids and skin organoids, successfully forming a fully functional vascularized skin organoid that produces multiple immune cell populations, but with a long differentiation period (>130 days), limiting its application. The synergistic action of vascular-immune-follicle improved the maturation efficiency of HFOs, which may become the basis for future research on human skin development, disease models, and reconstructive surgery (Qin et al., 2022).

The main content of the HFOs construction strategy is summarized in Table 1. The table shows that the hanging drop culture and Matrigel co-culture methods are easy to operate and have a relatively high short-term efficiency, making them suitable for the rapid formation of hair follicle buds; the ALI culture significantly enhances differentiation and accessory structure formation capabilities, but has a longer culture cycle and lower efficiency; while the directed differentiation method of iPSCs can construct more complex hair follicle-containing skin organoids, it faces challenges such as long culture time and high technical requirements.

**TABLE 1:** Comparison of major construction methods for hair follicle organoids.

Construction method	Cell source & ratio	Culture duration/Key time points	Formation efficiency & maturity indicators	Main advantages	Main limitations
Hanging Drop/Suspension Culture	Neonatal mouse epidermal: dermal cells (1:1–2:1)	24–48 h aggregation; 7–10 days maturation	Hair germ formation ∼70–90%; hair shaft ∼1–3 mm	Simple operation, low cost, rapid self-assembly	Poor size control, limited long-term stability, low vascularization
Matrigel-embedded Co-culture	Epithelial stem cells: DPCs (1:1–2:1)	24 h initial aggregation; 10–23 days	Hair shaft formation rate ∼80%; pigmented shafts observed	Strong EMI, supports core-shell structure and hair shaft growth	Batch variability, animal-derived matrix, potential immunogenicity
Air-Liquid Interface (ALI) Culture	Keratinocytes + fibroblasts or iPSC-derived cells	60–130 days (placode at ∼60 days)	Stratified epidermis + skin appendages; hair follicle-like structures	High differentiation potential, mimics skin surface environment	Low efficiency, long culture period, high cost, limited hair shaft elongation
iPSC-directed Differentiation	Human iPSCs → ectoderm + mesoderm	55–75 days (placode); ∼130 days (mature)	Complex skin organoids containing HF, sebaceous glands, neurons; *in vivo* hair outgrowth	Scalable, human-specific, multi-lineage differentiation	Extremely long protocol (>130 days), low efficiency, high cost, ethical concerns

### 4.5 Other emerging technologies

In addition to the core technical steps in the preparation of organoids, such as the regulation of cell proportional aggregation, the provision of physical scaffolds by the extracellular matrix, the induction of directional differentiation signals, and the *in vitro* culture of somatic cell reprogramming pluripotent stem cells, various engineered auxiliary technologies have also been introduced in recent years (Wang et al., 2023). Microfluidic technology, dynamic culture systems, bioprinting, mechanical stimulation and other auxiliary technologies have been used to improve the structural accuracy, functional maturity and controllability of organoids. Photobiomodulation (PBM) can regulate the proliferation and induction capabilities of DPCs and HFSCs, and improve the structure of the dermis (Chang et al., 2025). It is a potential supplement for HFOs. For detailed principles and advantages and disadvantages, please refer to Table 2.

**TABLE 2:** Engineering auxiliary techniques in organoids.

Technical name	Principle	Advantages	Disadvantages
Microfluidics (Sugiyama et al., 2024)	Microchannel controls fluids/signals, achieving dynamic infusion and monitoring	Simulate the spatial and temporal microenvironment; enhance vascularization/maturation; high-throughput screening; precise cutting to promote EMI.	High equipment complexity/high cost; high professional requirements; large batch-to-batch variability; potential cell damage
Dynamic Culture Systems (Zhang et al., 2025)	Computational fluid dynamics to enhance nutrient exchange and 3D growth	Improving nutritional metabolism; promoting uniform growth/differentiation; simulated microgravity (SMG); large-scale cultivation	Maintenance is complex and prone to contamination; induces cellular stress; and requires specialized equipment
Bioprinting (Chen et al., 2023)	Deposit bioink layer by layer to precisely construct 3D structuresADDIN.	Predetermined structure construction; high survival rate; multicellular integration; personalized regeneration	Limited resolution and may damage cells
Mechanical Stimulation (Nam et al., 2025)	Physical forces (such as stretching or shear) regulate the cytoskeleton and cell differentiation, simulating skin tension	Regulation of proliferation/differentiation; simulation mechanics; improvement of model maturity	Overstimulation injury; difficulty in parameter control; poor repeatability
Biomaterials (Kim et al., 2026)	Provide a support structure; serve as a substrate for cultivation	Biocompatibility; reduces foreign body reaction; supports for graft survival	Variation of animal origin; difficult mechanical matching; inaccurate degradation; high cost
Organ-on-a-Chip (Vera et al., 2021)	Multi-chamber structure, simulating organ interaction	Integrated dynamic interaction; high-fidelity simulation; supports toxicity testing	Multidisciplinary integration; high cost; incomplete immune simulation
Photobiomodulation (Roets, 2023)	Regulation of light stimulation	Non-invasive; low cost; easy to integrate	Complex parameters; potential phototoxicity
CRISPR/Cas9 gene editing (Su et al., 2025)	Precise gene editing	Improving stability; personalized modeling	Missed targets; ethical risks; low delivery efficiency; strict regulation

## 5 Application of hair follicle organoids

After several years of methodological exploration, the construction of hair follicle organoids has evolved from early simple co-cultures to directed differentiation based on iPSCs, core-shell structure design, and integration with auxiliary engineering technologies (Quílez et al., 2024). Some models have already achieved *in vitro* generation of hair shafts, pigmentation, and even cyclic changes. These technological advancements have laid a solid foundation for applying human HFOs to fundamental mechanism research, drug screening, and regenerative medicine therapies.

### 5.1 Research on disease mechanisms

Organoids have the advantages of short cycle and higher physiological correlation, and can accurately reproduce the multi-layer structure of human skin (including epidermis, dermis, hair follicles, sebaceous glands and other skin appendages). *In vitro* culture system, it can maintain the characteristics of cell lineage differentiation, physiological functions and genetic information for a long time, and even construct disease-specific organoid models carrying specific gene mutations (Zhu et al., 2026). It is closer to the real physiological state and pathological characteristics of clinical skin. HFOs show great potential and research value in elucidating the cellular molecular mechanisms of hair diseases and screening potential therapeutic targets (Lakeh and Shafiee, 2025).

Allmeroth et al. (2021) demonstrated through the use of HFOs that polyamines play a translation-independent role in regulating cell fate, and that spermidine/spermine N1-acetyltransferase can maintain the activity of HFSCs by reducing translation efficiency, providing a novel target for interventions against age-related hair loss. Li et al. (2025a) used iPSC-derived skin organoids containing hair follicles as an Enterovirus 71 (EV-A71) infection model and found that EV-A71 can cause hair follicle damage through the autophagy pathway and the integrin/Hippo-YAP/TAZ signaling pathway. They also identified an autophagy-related protein as a potential drug target, providing evidence for the pathological mechanisms and targeted therapy of virus-induced hair loss. Xiao et al. (2024) demonstrated the role of the neuroendocrine-immune axis in stress-induced alopecia by applying HFOs and found that blocking IL-18 and IL-1β signaling can reverse the consumption of HFSCs in the model, providing experimental evidence for targeted therapy of stress-induced alopecia. Kim et al. (2024) constructed a skin photo-damage model using iPSCs and confirmed that ultraviolet radiation can induce hair follicle structural damage and pathological changes. Exosome therapy has been shown to effectively alleviate inflammatory reactions and promote hair follicle regeneration. Li et al. (2025b) discovered that the COL6A3-CD44 pathway promotes the early maintenance of melanocytes, while the SEMA3C-NRP1 pathway regulates the migration of melanocytes to specific areas of the hair follicle, providing new evidence for the study of the mechanism of hair graying.

### 5.2 Drug development and screening

A high-throughput screening platform based on hair follicle organoids can rapidly evaluate the effects of candidate drugs on the hair follicle growth cycle, hair shaft formation, and related signaling pathways, while also effectively predicting potential toxic side effects of the drugs (e.g., cytotoxicity to follicular epithelial cells, inhibition of DPCs function), significantly enhancing the efficiency and success rate of drug development. Applying this platform in the early stages of drug development enables efficient screening of candidate drugs and formulations.

A variety of drug delivery systems have been reported to promote wound healing and hair follicle regeneration. Multidomain peptide hydrogels have significant antimicrobial activity and antioxidant capacity, which can significantly shorten the full-thickness wound healing process in mice, and there is more hair follicle regeneration at the edge and center of the wound (Hussain et al., 2021). Zhang’s team (Zhang et al., 2020) designed and prepared a highly bioactive bioceramic fibrous membrane containing quercetin and copper chelates. This fiber membrane can effectively release quercetin and copper ions, inducing proliferation, migration, and differentiation of skin- and hair follicle-related cells, thereby effectively accelerating burn wound healing and promoting hair follicle tissue regeneration.

Yoon et al. (2023) by treating hair follicles with a Wnt activator (KY19382). Found that it could activate the Wnt/β-catenin pathway to increase hair length in *ex vivo* hair follicles, and that the activation of the Wnt/β-catenin pathway increased hair length in *ex vivo* hair follicles, it can accelerate reepithelialization and neo-epidermogenesis in the early stage of skin wound healing, and has the potential to be developed as a new wound healing-hair follicle regeneration bifunctional agent. Kageyama et al. (2024) used HFOs to test cinnamic acid *in vitro* and observed that cinnamic acid significantly promoted the growth and maturation of hair follicle. The results provide an experimental basis for the development of hair products targeting the oxytocin receptor.

### 5.3 Regenerative medicine transplantation

HFOs has a structure and function similar to natural hair follicles, providing a new ideal source of grafting material for treating hair loss. At the same time, HFOs does not exist in isolation; the precise construction of ECM in its microenvironment is a key step in achieving long-term survival and functionality after transplantation. Organoid-related regenerative medicine transplantation techniques provide solutions for functional skin reconstruction and hair follicle regeneration. By optimizing transplantation strategies (such as microneedle injection, scaffold loading) and microenvironmental regulation (such as Wnt signaling pathway activation, improving vascularization). This is expected to achieve functional skin reconstruction of HFOs. Relevant research has made significant progress in in vivo experiments and clinical settings.

Wang et al. (2016) successfully regenerated functional sebaceous glands and hair follicles by transplanting epidermal stem cells from adult foreskin combined with skin-derived precursors into wounds, and the transplanted cells maintained their hair follicle-inducing capacity *in vivo* for a long time. Lee et al. (2020) transplanted iPSC-derived skin organoids to incision sites on the backs of nude mice, and after 1 month the organoids naturally unfolded and integrated into the native skin, forming hairs that could grow outward. This provides experimental evidence for an organoid replacement transplantation strategy for patients with extensive skin defects. Integrating bioactive nanofibers and incorporating stem cell spheroids, Huh et al. created implantable multifunctional biomaterials that modulate the multi-stage process of wound healing and improve the recovery of normal tissue structure (Huh et al., 2025). Elmaadawi et al. (2018) investigated the application value of HFSCs in alopecia areata and androgenetic alopecia. After *in vitro* expansion of HFSCs, autologous transplantation was performed into the affected scalp regions. Clinical evaluation after 6 months demonstrated significant improvement in hair density and growth status at the treated sites, with no serious adverse events reported. However, due to the small sample size in the study, further multicenter, large-sample research is needed to confirm its safety and efficacy (Liu et al., 2023).

## 6 Challenges of hair follicle organoids

First, organoids have a rapid growth rate. During the culture process, as the volume increases, the metabolism of some core cells is also restricted, which is prone to cause local microenvironment imbalances (such as acidic environments and hypoxic states). This inhibits their long-term functional maintenance and large-scale cultivation. The cultivation conditions required for HFOs vary significantly at different growth stages. During the development process, there will be significant differences in size, tissue structure, biological functions, and gene expression. The precise regulation and directed differentiation of the HFOs development process still require optimization.

Second, although HFOs have greatly improved in simulating the morphology and functions of internal organs compared to 2D cell models, they usually lack a complete basement membrane structure, and the types of appendages such as sweat glands, blood vessels, various immune cells, and nerve cells are incomplete. The limited maturity of the model will prevent it from accurately simulating physiological processes, which is a major bottleneck restricting the application of HFO.

**FIGURE 2:**
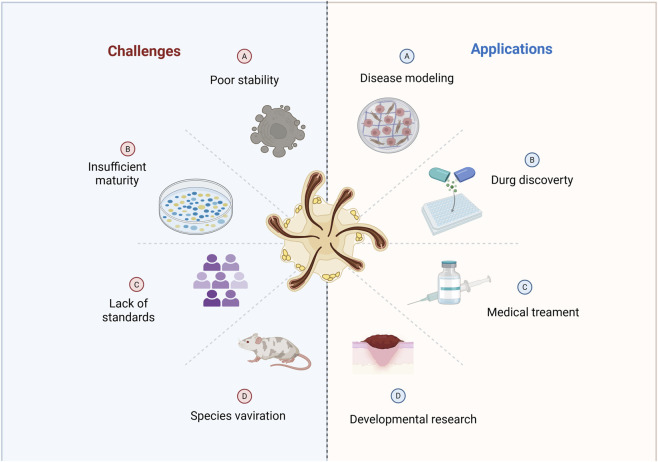
Applications and challenges of hair follicle organoids (HFOs). This schematic diagram summarizes the major applications and current technical challenges of hair follicle organoids. The central structure represents a hair follicle organoid. On the right (blue), key applications are illustrated: **(A)** disease modeling, **(B)** drug discovery, **(C)** medical treatment (regenerative transplantation), and **(D)** developmental research. On the left (red), major challenges are highlighted: **(A)** poor long-term stability, **(B)** insufficient structural and functional maturity, **(C)** lack of standardized protocols, and **(D)** species variation between mouse and human models. This figure emphasizes the need to overcome these limitations to bridge the gap between basic research and clinical translation of HFOs.

Third, there is a lack of uniform standards for the variables involved in culture, such as cell source, microenvironment construction and combination of growth factors. This may make it difficult to compare the results of different research teams horizontally, and there are challenges in reproducible preparation, which may affect the reliability and generalizability of research results. These situations all require systematic organization and evaluation. Please refer to Figure 2 for details.

Fourth, the differences accumulated during the embryonic development stage led to significant differences in the underlying biological characteristics of organoids constructed from mouse and human sources. Genes such as LGR5, which affect hair follicle stem cell development, are present in HFSCs of humans, pigs, and mice, but exhibit significant interspecies differences (Polkoff et al., 2022). The skin of mice is loosely attached, with a thin dermis and epidermis that exhibit a wavy, synchronized periodic cycle; in contrast, human skin is tightly attached to deep tissues, featuring a thicker dermis and more developed epidermal stratification, with hair follicle cycles not being synchronized. This means that organoid strategies validated in murine cell bodies as well as in mouse models, such as scaffold mechanical parameters, integration time windows, *etc.*, cannot be directly translated to human-derived organoid systems. During the process of translating research from animal models to human medicine, species-specific differences in cell sources represent a critical issue that cannot be overlooked (Gopee et al., 2024).

## 7 Discussion

In recent years, significant progress has been made in the development of hair follicle organoids (HFOs), providing valuable *in vitro* platforms for investigating hair follicle biology and related disorders. However, despite the successful reconstruction of basic follicular structures, substantial differences remain among current approaches in terms of reproducibility, maturation, and translational potential.

Among existing strategies, co-culture systems based on primary epithelial and mesenchymal cells can effectively recapitulate EMIs, which are essential for folliculogenesis. Nevertheless, their application is limited by donor variability and the rapid loss of inductive capacity during *in vitro* expansion. In contrast, iPSC-based approaches offer advantages in scalability and cell source accessibility, and enable the generation of complex skin appendages. However, these systems are often associated with prolonged differentiation timelines, relatively low efficiency, and incomplete structural and functional maturation. Therefore, achieving a balance between efficiency and physiological relevance remains a central challenge.

Importantly, although many studies have reported the formation of follicle-like structures, the long-term survival and functional integration of HFOs *in vivo* remain limited. Insufficient vascularization, the absence of nerve innervation, and the deficiency of the immune microenvironment all affect the regulation of the hair follicle cycle and its responsiveness to external stimuli. The single-cell type organoid system is difficult to fully simulate the real tissue environment. Therefore, it is necessary to achieve more complex tissue reconstruction through multi-cell co-culture or in combination with organ-on-a-chip technology.

From an application perspective, HFOs have demonstrated considerable potential in disease modeling and drug screening, particularly for alopecia, pigmentation disorders, and inflammatory skin diseases. However, most current studies remain at the preclinical stage, and their translational feasibility requires further validation. Future efforts should focus on establishing standardized protocols, including unified criteria for cell sourcing, culture conditions, and functional evaluation, to enhance reproducibility and comparability across studies.

In summary, HFOs represent a promising bridge between basic research and clinical application. The integration of emerging technologies, such as biomaterials, microfluidic systems, and bioprinting, is expected to facilitate the development of more physiologically relevant and functionally mature models, thereby accelerating their application in regenerative medicine and precision therapeutics.

## References

[B1] Allmeroth K. Kim C. S. Annibal A. Pouikli A. Koester J. Derisbourg M. J. (2021). N1-acetylspermidine is a determinant of hair follicle stem cell fate. J. Cell Science 134 (9), jcs252767. 10.1242/jcs.252767 33973637 PMC8182411

[B2] Bajouri A. Aghdami N. Nilforoushzadeh M. A. (2025). Synergizing biomaterials and cellular molecular pathways: a new frontier in hair follicle generation. J. Translational Medicine 23 (1), 1170. 10.1186/s12967-025-07211-w PMC12676848 41345670

[B3] Bauer M. Metzger M. Corea M. Schädl B. Grillari J. Dungel P. (2022). Novel 3D-Printed cell culture inserts for air-liquid interface cell culture. Life Basel, Switz. 12 (8), 1216. 10.3390/life12081216 PMC9410432 36013395

[B4] Burshtein J. Burshtein A. Schlesinger T. (2026). Emerging pharmacotherapies and regenerative solutions for promoting hair growth for androgenetic alopecia. Front. Pharmacology 17, 171776134. 10.3389/fphar.2026.1776134 PMC13033719 41919230

[B5] Cao X. Lu M. Li N. Cai L. Wang Y. Zhao Y. (2025). Emerging biomedical engineering strategies for hair follicle regeneration. Bioact. Mater. 53, 84–113. 10.1016/j.bioactmat.2025.06.051 40688016 PMC12274855

[B6] Ceruti J. M. Oppenheimer F. M. Leirós G. J. Balañá M. E. (2021). Androgens downregulate BMP2 impairing the inductive role of dermal papilla cells on hair follicle stem cells differentiation. Mol. Cellular Endocrinology, 520, 111096. 10.1016/j.mce.2020.111096 33259912

[B7] Chang H. Shen Q. Tan Y. Tong J. Zhang Z. Ouyang W. (2025). Red light promotes dermis-epidermis remodeling *via* TGFβ and AKT-mediated collagen dynamics in naturally aging mice. Zoological Research 46 (5), 967–982. 10.24272/j.issn.2095-8137.2024.405 40808593 PMC12780493

[B8] Chen H. Ma X. Gao T. Zhao W. Xu T. Liu Z. (2023). Robot-assisted *in situ* bioprinting of gelatin methacrylate hydrogels with stem cells induces hair follicle-inclusive skin regeneration. Biomed. and Pharmacotherapy = Biomedecine and Pharmacotherapie 158, 158114140. 10.1016/j.biopha.2022.114140 36535200

[B9] Corrò C. Novellasdemunt L. Li V. S. W. (2020). A brief history of organoids. Am. Journal Physiology. Cell Physiology 319 (1), C151–C165. 10.1152/ajpcell.00120.2020 32459504 PMC7468890

[B10] Cui C. Y. Kunisada M. Piao Y. Childress V. Ko M. S. H. Schlessinger D. (2010). Dkk4 and Eda regulate distinctive developmental mechanisms for subtypes of mouse hair. PloS One 5 (4), e10009. 10.1371/journal.pone.0010009 20386733 PMC2850388

[B11] de Groot S. C. Ulrich M. M. W. Gho C. G. Huisman M. A. (2021). Back to the future: from appendage development toward future human hair follicle neogenesis. Front. Cell. Dev. Biol. 9, 9661787. 10.3389/fcell.2021.661787 PMC8075059 33912569

[B12] Elmaadawi I. H. Mohamed B. M. Ibrahim Z. A. S. Abdou S. M. EL Attar Y. A. Youssef A. (2018). Stem cell therapy as a novel therapeutic intervention for resistant cases of alopecia areata and androgenetic alopecia. J. Dermatological Treatment 29 (5), 431–440. 10.1080/09546634.2016.1227419 27553744

[B13] Ge W. Tan S. J. Wang S. H. Li L. Sun X. F. Shen W. (2020). Single-cell Transcriptome Profiling reveals Dermal and Epithelial cell fate decisions during Embryonic Hair follicle development. Theranostics 10 (17), 7581–7598. 10.7150/thno.44306 32685006 PMC7359078

[B14] Gopee N. H. Winheim E. Olabi B. Admane C. Foster A. R. Huang N. (2024). A prenatal skin atlas reveals immune regulation of human skin morphogenesis. Nature 635 (8039), 679–689. 10.1038/s41586-024-08002-x 39415002 PMC11578897

[B15] Higgins C. A. Chen J. C. Cerise J. E. Jahoda C. A. B. Christiano A. M. (2013). Microenvironmental reprogramming by three-dimensional culture enables dermal papilla cells to induce *de novo* human hair-follicle growth. Proc. Natl. Acad. Sci. U. S. A. 110 (49), 19679–19688. 10.1073/pnas.1309970110 24145441 PMC3856847

[B16] Hu S. Li Z. Lutz H. Huang K. Su T. Cores J. (2020). Dermal exosomes containing miR-218-5p promote hair regeneration by regulating β-catenin signaling. Sci. Advances 6 (30), eaba1685. 10.1126/sciadv.aba1685 32832660 PMC7439409

[B17] Huh S. J. Han Y. Lee J. Park E. Lee S. H. Na J. (2025). Facile decellularization of stem cell spheroids by integrating bioactive nanofibers for enhanced wound healing. Small Weinheim der Bergstrasse, Ger. 21 (44), e06500. 10.1002/smll.202506500 40965241

[B18] Hussain M. Suo H. Xie Y. Wang K. Wang H. Hou Z. (2021). Dopamine-substituted multidomain peptide hydrogel with inherent antimicrobial activity and antioxidant capability for infected wound healing. ACS Applied Materials and Interfaces 13 (25), 29380–29391. 10.1021/acsami.1c07656 34128656

[B19] Ji S. Zhu Z. Sun X. Fu X. (2021). Functional hair follicle regeneration: an updated review. Signal Transduction Targeted Therapy 6 (1), 66. 10.1038/s41392-020-00441-y 33594043 PMC7886855

[B20] Kageyama T. Yoshimura C. Myasnikova D. Kataoka K. Nittami T. Maruo S. (2018). Spontaneous hair follicle germ (HFG) formation *in vitro,*, enabling the large-scale production of HFGs for regenerative medicine. Biomaterials 154291-300, 291–300. 10.1016/j.biomaterials.2017.10.056 29156398

[B21] Kageyama T. Chun Y. S. Fukuda J. (2021). Hair follicle germs containing vascular endothelial cells for hair regenerative medicine. Sci. Reports 11 (1), 624. 10.1038/s41598-020-79722-z PMC7804392 33436760

[B22] Kageyama T. Shimizu A. Anakama R. Nakajima R. Suzuki K. Okubo Y. (2022). Reprogramming of three-dimensional microenvironments for *in vitro* hair follicle induction. Sci. Advances 8 (42), eadd4603. 10.1126/sciadv.add4603 36269827 PMC9586475

[B23] Kageyama T. Seo J. Yan L. Fukuda J. (2024). Cinnamic acid promotes elongation of hair peg-like sprouting in hair follicle organoids *via* oxytocin receptor activation. Sci. Reports 14 (1), 4709. 10.1038/s41598-024-55377-y PMC10897452 38409197

[B24] Kageyama T. Anakama R. Hamano S. Tu S. Migita Y. Asaba T. (2026). Hair follicle organoids using human iPSC-Derived ectodermal precursor cells for hair regenerative medicine. ACS Biomaterials Science and Engineering 12 (3), 1704–1714. 10.1021/acsbiomaterials.5c01780 41664452 PMC12976999

[B25] Kang B. M. Kwack M. H. Kim M. K. Kim J. C. Sung Y. K. (2012). Sphere formation increases the ability of cultured human dermal papilla cells to induce hair follicles from mouse epidermal cells in a reconstitution assay. J. Investigative Dermatology 132 (1), 237–239. 10.1038/jid.2011.250 21850026

[B26] Kim M. J. Ahn H. E. J. Kong D. Lee S. Kim D. H. Kang K. S. (2024). Modeling of solar UV-induced photodamage on the hair follicles in human skin organoids. J. Tissue Engineering 15, 1520417314241248753. 10.1177/20417314241248753 PMC11080775 38725732

[B27] Kim J. S. Byun J. Choi J. Jin D. Li Q. Lee J. (2026). Cold atmospheric plasma-activated *in situ* hydrogel induces hair regeneration *via* immune microenvironment remodeling. Adv. Science Weinheim, Baden-Wurttemberg, Ger. 13 (5), e11962. 10.1002/advs.202511962 PMC12850053 41208290

[B28] Klingenstein S. Kleger A. Liebau S. Klingenstein M. (2025). State-of-the-Art: somatic cell sources used for the generation of human induced pluripotent stem cells. Stem Cells Development 34 (15-16), 317–332. 10.1089/scd.2025.0082 40601490

[B29] Lakeh B. Shafiee A. (2025). Advancing dermatology with skin equivalents and organoids in pathophysiology and drug testing. Acta Biomater. 207, 207120–207130. 10.1016/j.actbio.2025.10.008 41072597

[B30] Lee J. Bӧscke R. Tang P. C. A. O. Hartman B. H. Heller S. Koehler K. R. (2018). Hair follicle development in mouse pluripotent stem cell-derived skin organoids. Cell Reports 22 (1), 242–254. 10.1016/j.celrep.2017.12.007 29298425 PMC5806130

[B31] Lee J. Rabbani C. C. Gao H. Steinhart M. R. Woodruff B. M. Pflum Z. E. (2020). Hair-bearing human skin generated entirely from pluripotent stem cells. Nature 582 (7812), 399–404. 10.1038/s41586-020-2352-3 32494013 PMC7593871

[B32] Lee J. van der Valk W. H. Serdy S. A. Deakin C. Kim J. LE A. P. (2022). Generation and characterization of hair-bearing skin organoids from human pluripotent stem cells. Nat. Protocols 17 (5), 1266–1305. 10.1038/s41596-022-00681-y 35322210 PMC10461778

[B33] Li J. Ma J. Cao R. Zhang Q. Li M. Wang W. (2025a). A skin organoid-based infection platform identifies an inhibitor specific for HFMD. Nat. Communications 16 (1), 2513. 10.1038/s41467-025-57610-2 PMC11906866 40082449

[B34] Li T. Li X. Xiang X. Huang J. Shen X. Wang M. (2025b). Regenerative hair pigmentation *via* skin organoids: adaptive patterning mediated by collagen VI and semaphorin 3C. Adv. Science Weinheim, Baden-Wurttemberg, Ger. 12 (36), e02436. 10.1002/advs.202502436 PMC12462959 40611437

[B35] Liang L. Zhou J. Wang W. Wang W. Liu Y. Li J. (2026a). Spatially resolved proteomic mapping in skin organoid for hair follicle development. Mol. and Cellular Proteomics MCP 25 (1), 101482. 10.1016/j.mcpro.2025.101482 41380997 PMC12805098

[B36] Liang S. Qian Z. Wang Y. Huangfu J. Ren W. (2026b). From hPSCs to MSCs: differentiation strategies, pathways, and the emergence of common regulatory networks. Cell Mol. Biol. Lett. 31 (1), 43. 10.1186/s11658-026-00886-z 41735837 PMC13040787

[B37] Liu Z. Huang J. Kang D. Zhou Y. DU L. Qu Q. (2023). Microenvironmental reprogramming of human dermal papilla cells for hair follicle tissue engineering. Acta Biomater. 165, 16531–16549. 10.1016/j.actbio.2022.11.004 36347448

[B38] Liu C. Liu X. Li L. Wang W. Mi W. Gao S. (2026). Cystine promotes the development of hair follicle in rabbits by regulating wnt signalling pathway. J. Animal Physiology Animal Nutrition 110, 398–409. 10.1111/jpn.70050 41739955

[B39] Mcelwee K. J. Sundberg J. P. (2025). Innovative strategies for the discovery of new drugs against androgenetic alopecia. Expert Opinion Drug Discovery 20 (4), 517–536. 10.1080/17460441.2025.2473905 40029254

[B40] Mehta A. Motavaf M. Raza D. Mclure A. J. Osei-Opare K. D. Bordone L. A. (2025). Revolutionary approaches to hair regrowth: follicle neogenesis, Wnt/ß-Catenin signaling, and emerging therapies. Cells 14 (11), 779. 10.3390/cells14110779 40497955 PMC12153676

[B41] Mostina M. Sun J. Sim S. L. Ahmed I. A. Souza-Fonesca-Guimaraes F. Wolvetang E. J. (2025). Coordinated development of immune cell populations in vascularized skin organoids from human induced pluripotent stem cells. Adv. Healthcare Materials 14 (31), e02108. 10.1002/adhm.202502108 PMC12683213 40817680

[B42] Nakajima R. Tate Y. Yan L. Kageyama T. Fukuda J. (2021). Impact of adipose-derived stem cells on engineering hair follicle germ-like tissue grafts for hair regenerative medicine. J. Bioscience Bioengineering 131 (6), 679–685. 10.1016/j.jbiosc.2021.02.001 33678531

[B43] Nam S. Y. Jain S. K. Kurian A. G. Jeong I. Park B. C. Ban K. (2025). Hair regeneration: mechano-activation and related therapeutic approaches. J. Tissue Engineering 16, 1620417314251362398. 10.1177/20417314251362398 PMC12464413 41020043

[B44] Polkoff K. M. Gupta N. K. Green A. J. Murphy Y. Chung J. Gleason K. L. (2022). LGR5 is a conserved marker of hair follicle stem cells in multiple species and is present early and throughout follicle morphogenesis. Sci. Reports 12 (1), 9104. 10.1038/s41598-022-13056-w PMC9160037 35650234

[B45] Qin J. Chen F. Wu P. Sun G. (2022). Recent advances in bioengineered scaffolds for cutaneous wound healing. Front. Bioeng. Biotechnol. 10, 10841583. 10.3389/fbioe.2022.841583 PMC8921732 35299645

[B46] Quílez C. Jeon E. Y. Pappalardo A. Pathak P. Abaci H. E. (2024). Efficient generation of skin organoids from pluripotent cells *via* defined extracellular matrix cues and morphogen gradients in a spindle-shaped microfluidic device. Adv. Healthcare Materials 13 (20), e2400405. 10.1002/adhm.202400405 PMC11305970 38452278

[B47] Ramovs V. Janssen H. Fuentes I. Pitaval A. Rachidi W. Chuva De Sousa Lopes S. M. (2022). Characterization of the epidermal-dermal junction in hiPSC-derived skin organoids. Stem Cell Reports 17 (6), 1279–1288. 10.1016/j.stemcr.2022.04.008 35561682 PMC9213820

[B48] Roets B. (2023). “Potential application of PBM use in hair follicle organoid culture for the treatment of androgenic alopecia,” 23. 10.1016/j.mtbio.2023.100851 PMC10663892 38024838

[B49] Rogers G. Martinet N. Steinert P. Wynn P. Roop D. Kilkenny A. (1987). Cultivation of murine hair follicles as organoids in a collagen matrix. J. Investigative Dermatology 89 (4), 369–379. 10.1111/1523-1747.ep12471760 2822817

[B50] Sato T. Vries R. G. Snippert H. J. van de Wetering M. Barker N. Stange D. E. (2009). Single Lgr5 stem cells build crypt-villus structures *in vitro* without a mesenchymal niche. Nature 459 (7244), 262–265. 10.1038/nature07935 19329995

[B51] Su Y. Wen J. Zhu J. Xie Z. Liu C. Ma C. (2019). Pre-aggregation of scalp progenitor dermal and epidermal stem cells activates the WNT pathway and promotes hair follicle formation in *in vitro* and *in vivo* systems. Stem Cell Research and Therapy 10 (1), 403. 10.1186/s13287-019-1504-6 31856904 PMC6921573

[B52] Su R. Shen G. Xiao X. Zheng Y. Liu F. Chen D. (2025). Generation of a novel inducible and dermal papilla-specific Wif1-CreER Knock-In mouse line for hair follicle research. Exp. Dermatology 34 (5), e70109. 10.1111/exd.70109 40329691

[B53] Sugiyama E. Nanmo A. Nie X. Chang S. Y. Hashimoto M. Suzuki A. (2024). Large-scale preparation of hair follicle germs using a microfluidic device. ACS Biomater. Sci. Eng. 10 (2), 998–1005. 10.1021/acsbiomaterials.3c01346 38193447 PMC10865290

[B54] Sulic A. N. M. Das Roy R. Papagno V. Lan Q. Saikkonen R. Jernvall J. (2023). Transcriptomic landscape of early hair follicle and epidermal development. Cell Reports 42 (6), 112643. 10.1016/j.celrep.2023.112643 37318953

[B55] Sun J. Ahmed I. Brown J. Khosrotehrani K. Shafiee A. (2025). The Empowering Influence of air-liquid Interface Culture on Skin Organoid Hair Follicle Development, Burns and Trauma. 13tkae070. 10.1093/burnst/tkae070 PMC11736897 39822647

[B56] Takahashi K. Yamanaka S. (2006). Induction of pluripotent stem cells from mouse embryonic and adult fibroblast cultures by defined factors. Cell 126 (4), 663–676. 10.1016/j.cell.2006.07.024 16904174

[B57] Topouzi H. Logan N. J. Williams G. Higgins C. A. (2017). Methods for the isolation and 3D culture of dermal papilla cells from human hair follicles. Exp. Dermatology 26 (6), 491–496. 10.1111/exd.13368 28418608 PMC5519926

[B58] Vatanashevanopakorn C. Sartyoungkul T. (2023). iPSC-based approach for human hair follicle regeneration. Front. Cell. Dev. Biol. 11, 111149050. 10.3389/fcell.2023.1149050 PMC10266356 37325563

[B59] Vázquez-Ulloa E. Lin K. L. Lizano M. Sahlgren C. (2022). Reversible and bidirectional signaling of notch ligands. Crit. Rev. Biochem. Mol. Biol. 57 (4), 377–398. 10.1080/10409238.2022.2113029 36048510

[B60] Vera D. García-Díaz M. Torras N. Álvarez M. Villa R. Martinez E. (2021). Engineering tissue barrier models on hydrogel microfluidic platforms. ACS Appl. Mater. Interfaces 13 (12), 13920–13933. 10.1021/acsami.0c21573 33739812

[B61] Wang X. Wang X. Liu J. Cai T. Guo L. Wang S. (2016). Hair Follicle and Sebaceous Gland *de novo* Regeneration With Cultured Epidermal Stem Cells and Skin-Derived Precursors. Stem Cells Transl. Med. 5 (12), 1695–1706. 10.5966/sctm.2015-0397 27458264 PMC5189649

[B62] Wang M. Zhou X. Zhou S. Wang M. Jiang J. Wu W. (2023). Mechanical force drives the initial mesenchymal-epithelial interaction during skin organoid development. Theranostics 13 (9), 2930–2945. 10.7150/thno.83217 37284452 PMC10240816

[B63] Wang Z. Bai H. Wang S. Wang S. Yuan C. Ding Z. (2026). Targeting endogenous wnt antagonists for therapy development in AGA: a focus on DKKs and sFRPs. Stem Cell Reviews Reports 22, 1627–1640. 10.1007/s12015-026-11095-8 41779360

[B64] Weber E. L. Woolley T. E. Yeh C. Y. Ou K. L. Maini P. K. Chuong C. M. (2019). Self-organizing hair peg-like structures from dissociated skin progenitor cells: new insights for human hair follicle organoid engineering and turing patterning in an asymmetric morphogenetic field. Exp. Dermatology 28 (4), 355–366. 10.1111/exd.13891 30681746 PMC6488368

[B65] Worst P. K. Mackenzie I. C. Fusenig N. E. (1982). Reformation of organized epidermal structure by transplantation of suspensions and cultures of epidermal and dermal cells. Cell Tissue Research 225 (1), 65–77. 10.1007/BF00216219 6749296

[B66] Xiao X. Gao Y. Yan L. Deng C. Wu W. Lu X. (2024). M1 polarization of macrophages promotes stress-induced hair loss *via* interleukin-18 and interleukin-1β. J. Cellular Physiology 239 (4), e31181. 10.1002/jcp.31181 38219076

[B67] Yoon M. Kim E. Seo S. H. Kim G. U. Choi K. Y. (2023). KY19382 accelerates cutaneous wound healing *via* activation of the Wnt/β-Catenin signaling pathway. Int. J. Mol. Sci. 24 (14), 11742. 10.3390/ijms241411742 37511501 PMC10380997

[B68] Zhang Z. Dai Q. Zhang Y. Zhuang H. Wang E. Xu Q. (2020). Design of a multifunctional biomaterial inspired by ancient Chinese medicine for hair regeneration in burned skin. ACS Appl. Mater. Interfaces 12 (11), 12489–12499. 10.1021/acsami.9b22769 32118402

[B69] Zhang W. XI Y. Zhang H. Li X. Wang W. Zhao Z. (2025). Simulated microgravity induces time-dependent enhancement of *Pseudomonas aeruginosa* biofilm formation. Curr. Research Microbial Sciences 9, 9100493. 10.1016/j.crmicr.2025.100493 PMC12593438 41209717

[B70] Zhao B. Luo H. He J. Huang X. Chen S. Fu X. (2021). Comprehensive transcriptome and methylome analysis delineates the biological basis of hair follicle development and wool-related traits in merino sheep. BMC Biology 19 (1), 197. 10.1186/s12915-021-01127-9 34503498 PMC8427949

[B71] Zhou H. Zhang Y. X. Lei Q. K. Li Y. M. Zheng Y. W. (2025). Causes and therapeutic limitations of clinical alopecia and the advent of human pluripotent stem cell follicular transplantation. Stem Cell Research and Therapy 16 (1), 338. 10.1186/s13287-025-04447-7 40598627 PMC12220152

[B72] ZhuY. LiuS. WangC. XuZ. ShiZ. XingX. (2026). Effect of platelet-rich plasma in refractory vitiligo by promoting the differentiation of dermal melanocyte progenitors *via* IGFBP-2/RhoA/YAP pathway. J. Advanced Research S2090-1232 (26), 00191-8. 10.1016/j.jare.2026.02.057 41775322

